# The relationship between empathy and emotional intelligence among Iranian nursing students

**DOI:** 10.5116/ijme.5b83.e2a5

**Published:** 2018-09-19

**Authors:** Fatemeh Hajibabaee, Mansoureh A. Farahani, Zahra Ameri, Tahmineh Salehi, Fatemeh Hosseini

**Affiliations:** 1 Nursing and Midwifery School, Tehran University of Medical Sciences, Iran; 2 Nursing Care Research Center, Iran University of Medical Sciences, Iran; 3 School of Nursing and Midwifery, Shahid Beheshti University of Medical Sciences, Iran; 4 School of Nursing and Midwifery, Iran University of Medical Sciences, Iran; 5 School of Health Management and Information Sciences, Iran University of Medical Sciences, Iran

**Keywords:** Empathy, emotional intelligence, nursing students, education

## Abstract

**Objectives:**

To determine the relationship between empathy and emotional intelligence among Iranian nursing students.

**Methods:**

This is a cross-sectional, descriptive-correlational study that was conducted on three hundred and twenty eligible students, selected using stratified random sampling. Participants were mainly nursing students at Tehran University of Medical Sciences. Data gathering was done using The Jefferson Scale of Empathy and The Schutte Self Report Emotional Intelligence Test. Data were analyzed using SPSS.

**Results:**

The results showed a strong positive correlation between empathy and emotional intelligence (r=0.499, p <.001). Students in their fourth year had the highest score for empathy (M=109.16, SD=10.16), while first-year students had the highest scores for emotional intelligence (M= 151.68, SD= 17.47). Female students got higher empathy scores than male students (t_(318)_= 2.524, p= .012). Age had a strong inverse correlation with emotional intelligence (r= 0.143, p= .010).

**Conclusions:**

The results of this study show a correlation between emotional intelligence and empathy among nursing students. Nurses with higher emotional intelligence tend to be better in establishing productive relationships with patients and their families, and if nurses possess empathetic skills, they manage their emotions more effectively. In addition to imparting knowledge and clinical experience, nursing curricula should provide students with opportunities to develop their communication and emotional skills.

## Introduction

Empathy is the ability to understand and experience other people’s feelings,^1^ and in the healthcare setting, it can be divided into three components: the sharing of a patient’s emotional state; the explicit understanding of a patient’s emotional state; and the prosocial behaviors that follow.^2^^,^^3^ Empathy is a skill that is necessary while managing relationships. However, studies have shown that nursing students often find it challenging to manage both personal and interpersonal relationships, especially with patients. College years are very stressful, and students have to grapple with a variety of problems during this period.^4^

Emotional intelligence is made up of a set of skills that lead to a better understanding of one’s own feelings and that of others.^5^ It is defined as the ability to identify and recognize the meanings and concepts of emotions, the relationships between them, the reasons behind them, problem-solving based on them, and how to manage emotions.^6^

Emotional intelligence forms an important part of nurses’ clinical practice. Through emotional intelligence, nurses learn how to deal with their feelings, as well as provide emotional support to patients and their families in multi-dimensional clinical environments. Emotional intelligence is equally important in developing individual’s decision-making skills and problem-solving abilities, thereby generally improving the performance of nurses.^7^^,^^8^^,^^9^

Nurses who possess a sufficient level of emotional intelligence are well placed to provide services in a way that improves patient satisfaction. They tend to understand interpersonal messages better, have better listening skills, and demonstrate more insight than their counterparts without such capabilities. Also, they are more skilled at managing their own emotions.^5^

Por and colleagues opined that students with higher emotional intelligence could manage their emotions, are more informed of their feelings, and most likely to enjoy good health. Moreover, people with higher emotional intelligence scores tend to have more advanced social abilities, richer social forms of communication, and more effective coping strategies.^10^

Studies have shown that people’s empathy and emotional intelligence skills change during educational years.^11^ A study conducted among medical students found that their levels of empathy are inversely related to the number of years of their education. The mean score of empathy was 97.4 for junior dentistry students, and 85.97 for senior students and residents, suggesting a trend of lower empathy scores, depending on the number of years they have studied.^12^

In another study, Benson and colleagues found that senior nursing students had higher empathy scores than freshman students. They also reported that 24 percent of fourth-year nursing students demonstrated an enhanced level of emotional intelligence and well-developed emotional and social capacities.^13^ Furthermore, Austin and colleagues found a significantly positive correlation between empathy and emotional intelligence among first-year medical students.^14^

A number of studies have been conducted on emotional intelligence and empathy among healthcare professionals, including nurses and nursing students.^13^^,^^15^^,^^16^ In order to enhance the quality of care that nurses provide to patients, it is important to identify various factors that can improve effective communication and empathetic abilities of nursing students, whom eventually will become nurses. Therefore, this study aimed to determine the possible correlation between empathy and emotional intelligence among nursing students.

## Methods

### Study design and participants

A cross-sectional, descriptive correlational study was conducted among nursing and midwifery students at Tehran University of Medical Sciences. Three hundred and twenty eligible participants were selected using stratified random sampling. Participants were selected from each year group. This was achieved by dividing the number of students in a particular year group by the total number of university students.

The inclusion criteria were: 1) being a nursing student who had completed at least one semester; 2) having enrolled for a clinical course; 3) having experience of communication with patients; and 4(willing to participate in this study. The minimum sample size was determined using the following parameters: an alpha of 0.05, a power of 0.80, and the results of an earlier pilot study. The pilot study was conducted among 40 nursing students who found a correlation coefficient between empathy and emotional intelligence (r = 0.361).

This study was approved by the Ethics Committees of the Tehran University of Medical Sciences. We gained formal permission from the relevant authorities in the School of Nursing and Midwifery, Tehran University of Medical Sciences. Moreover, we provided the participants with information about the aims and the importance of the study and allowed them to opt to participate voluntarily. Confidentiality of participants was respected, and study data were protected. Then, verbal and written informed consent was secured from all who were willing to participate.

### Data collection

Data was gathered using basic demographic questionnaire and standard scales such as; The Jefferson Scale of Empathy, and the Schutte Self-Report Emotional Intelligence Test. The Jefferson Scale of Empathy was developed in 2001 at the Jefferson medical school and consists of 45 items. It was designed based on the original scale in 2009 by Hojat and colleagues and then reduced to 20 items. The responses to each item are scored on a Likert scale ranging from strongly agree (7) to strongly disagree (1), with scores ranging from 20 to 140, with higher scores indicating higher empathy. The tool consists of three components – perspective-taking, compassionate care, and standing in the patient’s shoes – and has ten questions (1-3-6-7-8-11-12-14-18-19) scored in reverse order.^17^

The Schutte Emotional Intelligence test was developed in 1998 by Schutte and colleagues.^18^ The initial version had 33 items but due to the lack of inverse questions and the probability of bias, it was revised, and the number of items increased to 41. The 21 questions of the questionnaire are scored inversely; each item is answered on a 5-point Likert scale from strongly agree (5) to strongly disagree (1). It has a range of scores from 41 to 205, and the scale consists of three domains: emotion evaluations (40, 39, 36, 31, 28, 22, 17, 8, 6), emotion regulation (38, 37.35, 30, 29, 21, 18, 15, 12, 2), and emotion use (34, 26, 25, 23, 10, 9, 4).^19^

The validity of the Jefferson Scale of Empathy for nursing students was examined in a study with 333 nursing students conducted in 2009,^20^ finding a reliability coefficient of 0.77. We used a forward-backward translation method to translate the English version of the scale into Persian by two interpreters fluent in both English and Persian. In the present study, content validity was used to validate the Persian version of the empathy scale, and two translators fluent in both languages conducted a backward Persian-to-English translation. We sent the back-translated version to the scale developer who approved the translation. The English version of the scale was then translated into Persian by two interpreters fluent in both English and Persian, and the content validity was used to validate the Persian version of the empathy scale. The final version was given to ten faculty members of the School of Nursing and Midwifery, who were asked to rate each item based on relevance, clarity, simplicity, and ambiguity. After applying the experts’ opinion and comments, the final version of the scale was used.

The psychometric properties of the Persian version of the Schutte Emotional Intelligence Scale were determined in Besharat’s21 study of 442 students; he reported that the Persian version had a high test-retest reliability (r= 0.75) and internal consistency (α= 0.89).^21^ The test-retest reliability and internal consistency of the scale were investigated for the present study, and a Pearson’s correlation coefficient and Cronbach’s alpha were calculated at 0.80 and 0.85, respectively.

### Procedure

The participants were recruited from classrooms and practical training centers in hospitals during their internships. The first author introduced herself to the students and explained the aims of the study to them. She then asked for their voluntary participation and obtained informed consent from interested students. Questionnaires were then given out, which they spent between 20 and 25 minutes to complete and returned to the researcher.

### Data analysis

Data analysis was carried out in SPSS for Windows, Version 18. Data were analyzed using descriptive and analytical statistics, including frequency, mean, standard deviations, independent t-test, one-way analysis of variance (ANOVA), and Pearson’s correlation coefficient.

**Table 1:** Demographic characteristics of participants (N=320)

Demographic characteristics	Status	N	%
Sex	Male	97	30.3
	Female	223	69.7
Marital status	Single	285	89.1
	Married	35	10.9
Age	<20	116	36.3
	20-24	190	59.4
	≥25	14	4.3
Interested in nursing	Yes	229	71.6
	No	91	28.4
Employment status	employed	70	250
	unemployed	21.9	78.1
Lodging	Living in a dormitory	100	31.3
	Living in Tehran with family	167	52.4
	Rental home	9	2.8
	Living in environs of Tehran with family	41	12.9
	Other	2	0.6
Previous semester GPA	11-14.99	17	5.3
	15-16.99	130	40.6
	≥17	173	54.1

## Results

Most participants (59.4%) were aged 20-24 years, single (89.1%), unemployed (78.1%), and interested in nursing (71.6%). More than half (54.1%) had a Grade-Point Average (GPA) higher than 17 out of 20 and lived in Tehran with their families (52.4%). A great number of the participants (81.6%) had not attended any workshops related to emotional intelligence or effective communication (84.1%). Table 1 shows details of the participant’s characteristics.

**Table 2:** Comparing average scores of empathy and emotional intelligence in terms of years of university education

University education Year	Empathy Mean (SD)	Emotional intelligence Mean (SD)
First	100.85 (8.16)	151.68 (17.47)
Second	100.12 (10.77)	151.45 (14.48)
Third	103.62 (14.5)	146.83 (17.12)
Fourth	109.16 (10.61)	148.14 (17.80)
ANOVA test	F _(3, 316)_ =10.641 p<.001	F_ (3, 316)_ =1.563 p=.198

Participants in their fourth year had the highest score of empathy (F_(__3, 316)_=10.641, p<.001). Those in their first-year had the highest scores for emotional intelligence (M=151.68, SD= 17.47), while the fourth-year group had lower emotional intelligence scores (M=146.83, SD=17.12), see Table 2. The difference in the participant’s level of study and emotional intelligence scores was not statistically significant.

The independent sample t-test showed a statistically significant difference between female and male participants in terms of empathy (t_(__318)_= 2.524, p= .012), female participants had higher empathy scores than the males. There was also a strong inverse correlation between age and emotional intelligence (r= 0.143, p= .010). The results showed a strong positive correlation between empathy and emotional intelligence, indicating that with an increase in emotional intelligence scores, empathy scores also increase (r=0.499, p<.001).

## Discussion

This study sought to determine the relationship between empathy and emotional intelligence among nursing students. The results show a positive correlation between empathy and emotional intelligence—for every increase in score for empathy, emotional intelligence scores increase as well. Also, we found that empathy scores increased with the number of years the participants had spent at university. One conclusion seems to be that students’ clinical experience can be a decisive factor in high empathy scores. Contrary to our findings, Ward and colleagues found that empathy scores decrease with increases in the number of college years.^22^ This difference in results could be due to differences in educational methods and the nursing curriculum in the two respective settings in which the studies were conducted. In a study by Jabbarifar and colleagues, medical students in junior years achieved higher empathy scores than medical residents.^12^ The most important difference between the current study and that of Jabbarifar and colleagues is the difference between their study populations. The latter study was conducted among medical students, and it seems that the process of formation of empathy among medical students is different from that of nursing students. Other possible reasons for this difference is the increased need for technical skills in the final years of university education for medical students. Also, fatigue and increased stress levels, and the change in position from studentship to residency, and the fact that medical education does not require so much attention being paid to patients.^12^ The empathy scores found in this study were consistent with those found by Shariat and Kaikhavi who noted that empathy scores increased based on the number of years of university education, with fourth-year students revealing higher empathy scores than their first-year counterparts.^23^

A comparison of the emotional intelligence scores of students in different year levels at a university found that students in the first and third years had the highest and lowest scores of emotional intelligence and empathy respectively. Benson and colleagues conducted a study among 100 nursing students using the Bar-On Emotional Intelligence Scale. They found that fourth-year nursing students had the highest scores of emotional intelligence and that emotional intelligence scores increase as the students progress at university.^13^ This difference between our findings and those of studies conducted in Western countries may be due to differences in the cultural context. Since the professional position of nurses in Western countries is higher than that of nurses in developing countries such as Iran, it is likely to affect the motivation of nursing students. There are also a number of social and cultural factors that can reduce students’ motivation during their training, and that could also affect their emotional intelligence abilities. Therefore, advances should be made in encouraging and supporting students to apply and build their emotional intelligence skills. The inclusion of such courses in the curriculum is highly recommended. Workshops and training courses on emotional intelligence should also be held frequently among students while at university and after graduation.

The results of the current study indicate a higher empathy score on average for female students than for male students. Ward and colleagues’ study of 214 nursing students at Thomas Jefferson University found that empathy scores of female students were higher than those of male students, which is consistent with the results of the current study.^22^ In another study by Jabbarifar and colleagues on empathy among dental students, the mean and standard deviation of empathy scores were found to be (M= 88.93, SD= 9.50) and (M= 88.25, SD= 7.92) for female and male students respectively. This is consistent with the findings of this current study, in which female students had higher empathy scores.^12^ In Shariat and Keikhavi’s study of 251 medical residents at hospitals affiliated to Iran University of Medical Sciences, the authors used the Jefferson Empathy Scale (for physicians) and found that female residents had higher empathy scores than males, which is also consistent with our findings.^23^

Our findings show a significant relationship between empathy and emotional intelligence among nursing students. This is consistent with findings reported by Austin and colleagues which they found a positive correlation between emotional intelligence and empathy among students.^14^ These findings indicate that in addition to a good understanding of interpersonal messages, people with higher emotional intelligence have better empathetic skills, and better at organizing their emotions.^10^ Empathy is an important issue in modern moral philosophy, and the concept plays a central role in moral and social growth. It is also recognized as a key element for effective care, helping to increase patient satisfaction with the care process. One of the components of empathy is one’s emotional response to the feelings of other people. Emotional intelligence is related to the role of emotions and feelings in humans, and the development of emotional intelligence is a good strategy in the growth of nurses’ emotions and the establishment of empathetic relationships. Empathy and emotional intelligence are a set of skills that give people better understanding of their feelings and that of others. Therefore, more attention should be paid to the development of these skills, along with their key role in decision-making and problem-solving, in the design of curricula for nursing students.

### Limitations

A significant limitation of this current study was the self-reporting style of the questionnaires. Biased findings may have resulted due to the self-reported nature of the study. Another limitation worth noting is the fact that the study was conducted in a single institution, which constrains the generalizability of its findings to similar contexts. Thus, further and more extensive studies are recommended in different disciplines.

## Conclusions

The results of this study indicate a correlation between emotional intelligence and empathy among nursing students. Age and sex were found to be important factors in the development of these characteristics. Nurses with higher emotional intelligence tend to be better in establishing productive relationships with patients and their families, and if nurses possess empathetic skills, they manage their own emotions more effectively. In the complex health service environment, nurses who can communicate and control their feelings better are typically more successful. In addition to imparting knowledge and clinical experience, nursing curricula should provide students with opportunities to develop their communication and emotional skills. Emotional intelligence exerts a strong influence on nurses’ ability to establish empathetic relationships with patients, and empathetic nursing students are more likely to become effective members of healthcare teams who have a direct impact on patients’ well-being. Adding training courses that cover emotional control and empathy skills to the nursing curriculum, to be taught alongside other academic skills, could be an important approach. The results of this study also indicate that nursing education curriculum in Iran is not successful in the development of emotional intelligence among nursing students, and further studies are required to determine the cause of this.

### Acknowledgments

The authors wish to thank all of the nursing students for their participation in this study.

### Conflict of Interest

The authors declare that they have no conflict of interest.
